# Improving hand hygiene performance in a surgical ICU: a novel method for data collection to change physician behavior

**DOI:** 10.1186/cc11991

**Published:** 2013-03-19

**Authors:** SA Nasraway

**Affiliations:** 1 Tufts Medical Center, Boston, MA, USA

## Introduction

ICU-acquired infection is directly related to hospital mortality. Hand hygiene is an effective, low-cost intervention that can prevent the spread of bacterial pathogens, including multidrug-resistant organisms. Historical compliance with hand hygiene guidelines by physicians, nurses and other care providers is poor.

## Methods

Present expectations by the Infection Control Committee are to 'pump in, pump out' of every room, using 63% isopropyl alcohol. We performed 17,622 observations of hand hygiene in the surgical ICU from March through October 2012, and intervened to change behavior by providing monthly feedback to specific provider groups and services. We made use of the Unit Coordinator to measure compliance of all individuals in the ICU.

## Results

**Figure 1:**
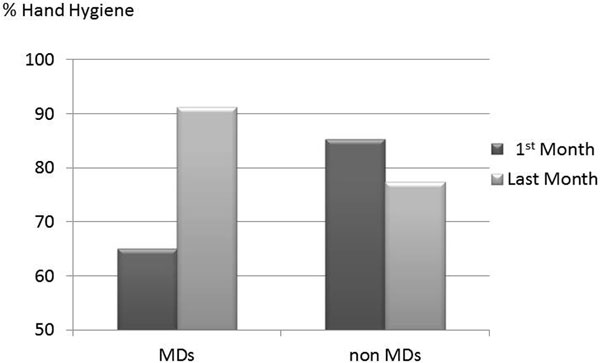


Overall compliance by physicians was 82.1%, for nonphysicians was 84.8%. Feedback to physicians, individually and by service, dramatically increased hand hygiene compliance, defined as both on entry and exit from the patient room, over the study period. See Figure 1.

## Conclusion

Physician behavior is responsive to monthly feedback that is specific to the individual or surgical service. Use of the Unit Coordinator was very effective at gathering a very large sample size in a short period of time.

